# Asphyxiated by a Tooth

**Published:** 1882-10

**Authors:** N. Miller

**Affiliations:** Preston.


### ARTICLE. V.

### *Asphyxiated by a Tooth.*

BY N. MILLER, L. D. S. I., PRESTON.

The following may not be without interest to many of your readers: A gentleman brought his son (a strong, healthy boy of between ten and eleven years of age) for consultation as to the state of his teeth. Nine of his temporary teeth obstructed the eruption of the permanent ones. This being explained to the father, he wished gas administered and the extraction of what was necessary. The boy, not being the least nervous, inhaled gas freely and became unconscious in from fifteen to twenty seconds; seven of the temporary teeth were extracted, the last being a left lower molar; during the latter stage of the operation the gag slipped and the mouth closed. The patient became partly conscious, assumed a natural color, when he took a deep inspiration, immediately after which he exhibited symptoms of asphyxia, raised his hand to his neck and attempted to tear his garments away, although they were quite loose. Immediately his head was held forward across the knee and some sharp raps given on his back, but it seemed to be of no avail; trial was then made to fell the tooth, but did not succeed. Seeing there was no improvement, a medical man was at once telephoned for, but must have been out, as no answer was received. Then the nearest medical man was sent for, and Dr. Marshall arrived in about seven minutes, who, when he saw the boy, pronounced life to be extinct.

A wish was expressed to the father that he would consent to the doctor performing tracheotomy, which he readily did. Dr. Marshall made a *post mortem* in the presence of myself and deceased's uncle, when he found the left lower molar, with roots uppermost, firmly fixed in the larynx.

An inquest was held the same evening, when the jury came to the unanimous conclusion that the cause of death was purely accidental. The father being asked if he attached any blame to any one, expressed himself perfectly satisfied with the course that had been pursued during the unhappy occurrence.

Thinking it would be better from a practical point of view if all accidents of an alarming nature were recorded in your valuable journal, for the benefit of those who may not have had such unpleasant experience, I send this account in the hope that it may act as a warning.

I cannot but think that amongst so many as there are in our profession many less serious accidents occur, the particulars of which would be interesting and instructive to all, except, perhaps, the parties immediately concerned, and would show in what proportion accidents happen, each teaching its own lesson.—*British Journal of Dental Science.*

